# Fluorescence Localization and Comparative Ultrastructural Study of Periplocoside NW from *Periploca sepium* Bunge in the Midgut of the Oriental Amyworm, *Mythimna separata* Walker (Lepidoptera: Noctuidae)

**DOI:** 10.3390/toxins6051575

**Published:** 2014-05-14

**Authors:** Mingxing Feng, Juan Zhao, Jiwen Zhang, Zhaonong Hu, Wenjun Wu

**Affiliations:** Institute of Pesticide Science, Northwest Agriculture and Forestry University, Yangling, Shaanxi 712100, China; E-Mails: fengmx2010@126.com (M.F.); zhaojuanaa2006@163.com (J.Z.); nwzjw@163.com (J.Z.); wuwenjun@nwsuaf.edu.cn (W.W.)

**Keywords:** periplocoside NW (PSNW), *Mythimna separata*, fluorescence localization, ultrastructure change, *Agrotis ispilon*, *Periploca sepium* Bunge

## Abstract

Periplocoside NW (PSNW) is a novel insecticidal compound isolated from the root bark of *Periploca sepium* Bunge and has potent stomach toxicity against some insect pests. Previous studies showed that the *Mythimna separata* larva is sensitive to PSNW, but the *Agrotis ispilon* larva is insensitive. In this study, preliminary target localization on the midgut of *M. separata* larvae was conducted via a fluorescence labeling technique. A comparative ultrastructural study on the effects of PSNW on the midguts of *M. separata* and *A. ispilon* larvae was performed. Symptom observation results showed that typical stomach toxicity was induced by PSNW in *M. separata* larvae. Fluorescence localization results showed that PSNW binds to the midgut cells of *M. separata* larvae. Ultrastructure observations showed destruction of the microvilli, organelle, and cytomembrane in the midgut cells of *M. separata* larvae, whereas no obvious changes were observed in midgut cells of *A. ispilon* larvae*.* These results were consistent with the insecticidal activity of PSNW. Therefore, PSNW might act on the midgut tissues of the insects, and one or more binding sites of PSNW may exist in *M. separata* larvae midgut cell cytomembranes.

## 1. Introduction

Increasing human health and environmental concerns propel the development of new natural pesticides, showing characteristics of specificity, safety to non-target organisms, particularly mammals and human, environmental friendliness, high effectiveness, and easy degradation [1]. Conventional chemical pesticides potentially show toxic effects in humans. For example, organophosphates (OPs) and carbamates-based insecticides are synaptic poisons by binding to and inhibiting acetylcholinesterase. Epidemiological studies suggest that exposure to pesticides may cause Parkinson’s disease in humans [2]. Conversely, botanical pesticides are generally less harmful than conventional pesticides and are important in specific integrated pest management strategies; these pesticides are very effective when produced and delivered correctly [3]. In line of the scope of this report, research and development of new pesticides involves discovering new insecticidal compounds from plant secondary metabolites and using these compounds as the primary components for further pesticide modification [4].

The root bark of *Periploca sepium* Bunge, a traditional Chinese herbal medicine from the Asclepiadaceae plant family, has been widely used in the treatment of autoimmune diseases, especially rheumatoid arthritis [5,6]. Periplocoside NW (PSNW) is a newly discovered pregnane glycoside component of *P. sepium* Bunge secondary metabolites that showed obvious insecticidal activity against several insect species [7]. PSNW has a special mode of action that involves stomach toxicity, but has no contact toxicity [8]. The Oriental Armyworm *Mythimna separata* is a severe pest of cereal crops, especially wheat, maize, and rice, throughout eastern China [9]. Currently, it is used widely as a standard test insect in modern laboratories for various research purposes.

Previous studies on PSNW have been conducted, but information on its mechanism of action and action location in *Mythimna separata* larvae is lacking. According to our previous study, PSNW shows stomach toxicity in *M. separata* larvae, but not on *Agrotis ispilon* (Hufnagel) larvae [8]. Therefore, the present study was conducted to determine its localization in the midgut of *M. separata* larvae. A comparative ultrastructural study was performed between *M. separata* and *A. ispilon* larvae after treatment with PSNW. The results will establish a foundation for further research on the mechanism of action and target localization of PSNW in agricultural pests.

## 2. Results

### 2.1. Symptom Observations

**Figure 1:**
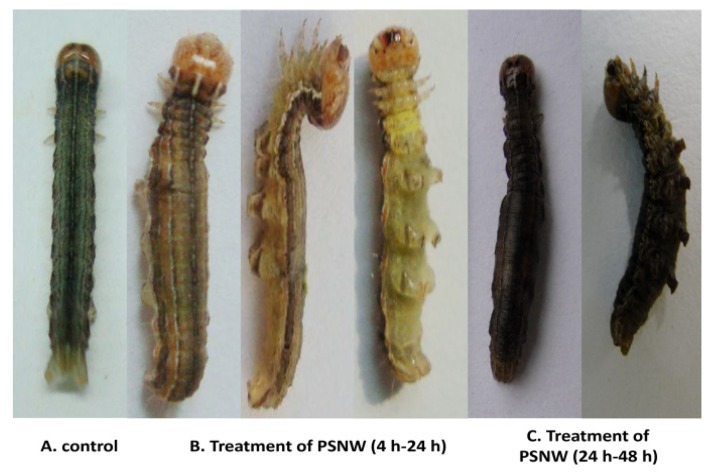
Intoxication symptoms induced by periplocoside NW (PSNW) in *M. separate* larvae.

Four hours after 5^th^ instar *M. separata* larvae were fed with a diet containing 5 µg PSNW, intoxication responses were observed. PSNW poisoning symptoms include cessation of feeding and swelling of the abdomen after 6 to 8 h. After 8 to 12 h, the insects were placed on the substrate in a prostrated orientation to avoid climbing behavior or body curving. The head and prosoma body of intoxicated insects were allowed to move freely. Twenty-four hours after exposure death occurred and the mortality rates reached more than 90%. The swollen abdomen darkened in color at the time of death (Figure 1). Control insects continued feeding and showed considerable growth by the end of the three-day assessment period. *A. ispilon* larvae did not show poisoning symptoms after undergoing the same procedure.

### 2.2. Fluorescence Localization of PSNW in the Midgut of M. separata Larvae

**Figure 2:**
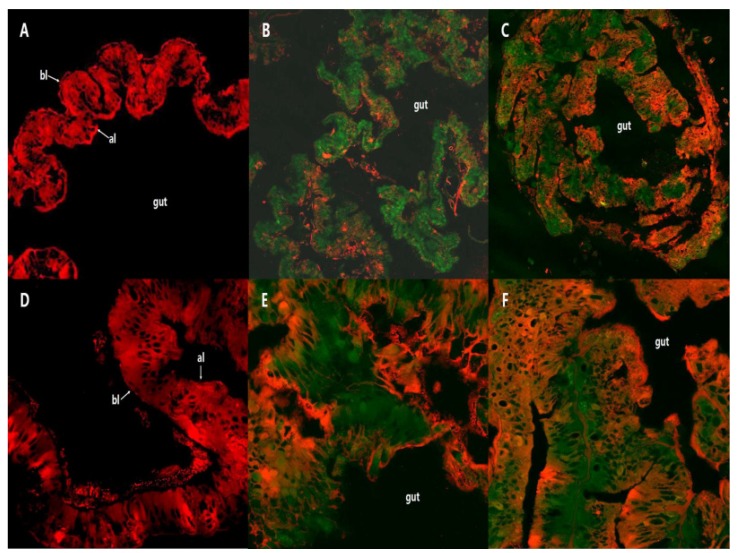
Fluorescence localization of periplocoside NW (PSNW) in the midgut of *M. separata* larvae. (**A**,**D**) Control midgut; (**B**,**E**) Incubated with fluorescence marker of PSNW (FNW) *in vivo*; (**C** and **F**) Incubated with FNW *in vitro*. A, B, and C, 100×; and D, E, and F 400×.

Confocal laser scanning microscopic (CLSM) observation showed that the structure of the cell plasma membrane in the *M. separata* larvae midgut displayed red fluorescence and the fluorescence marker of PSNW (FNW) in the *M. separata* larvae midgut tissue displayed green fluorescence. As shown in the figures, no emitted green fluorescence was found in the midgut of control *M. separata* larvae (Figure 2a,d), whereas the necrotic midgut tissue of *M. separata* larvae fed with wheat leaf containing FNW (*in vivo*) strongly emitted green fluorescence (Figure 2b,e). The midgut of the *M. separata* larvae incubated with FNW *in vitro* also emitted a relatively strong green fluorescence (Figure 2c,f).

The midgut of control larvae displayed intact structures and a clear profile, and showed homogeneous thickness (Figure 2a) and an intact cell plasma membrane (Figure 2d). Compared with the control, the *M. separata* larvae fed with wheat leaf spiked with FNW showed a damaged midgut (Figure 2e) with disarranged midgut cells. The cytoplasmic membrane was severely damaged or lost its integrity, and a cavity was observed in the cytoplasm (Figure 2e). Furthermore, midgut tissue in the *M. separata* larvae incubated with FNW *in vitro* showed the same phenomena (Figure 2c,f).

### 2.3. Comparative Ultrastructural Study of the Effect of PSNW on the Midgut of M. separata Larvae and A. ispilon Larvae

#### 2.3.1. Ultrastructure Effects of PSNW on Midgut Cells of *M. separata* Larvae

**Figure 3:**
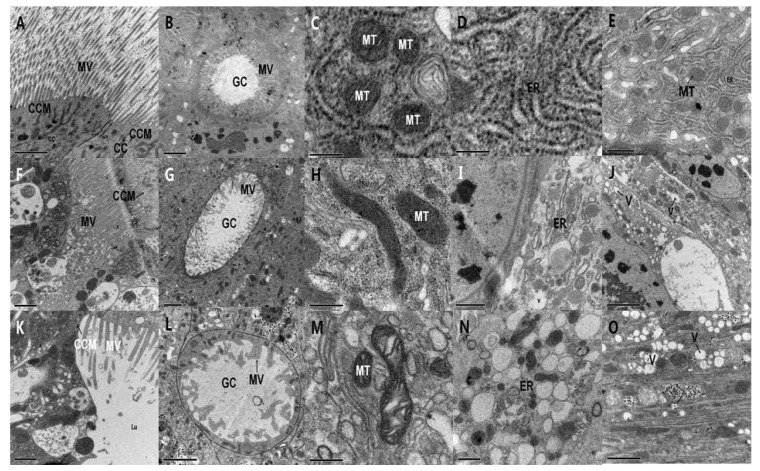
Ultrastructural effect of periplocoside NW (PSNW) on the midgut cells of *M. separata* larvae. (**A**) Control of columnar cell membrane and microvilli (15000×); (**B**) Control of goblet cells (12,000×); (**C**) Control of mitochondria (80,000×); (**D**) Control of endoplasmic reticulum (80,000×); (**E**) Control of cytoplasm (50,000×); (**F**) Columnar cell membrane and microvilli 10 h after treatment (20,000×); (**G**) Goblet cells 6 h after treatment (10,000×); (**H**) Mitochondria 8 h after treatment (60,000×); (**I**) Endoplasmic reticulum 6 h after treatment (25,000×); (**J**) Cytoplasm 6 h after treatment (30,000×); (**K**) Columnar cell membrane and microvilli after 6 h treatment (20,000×); (**L**) Goblet cells 6 h after treatment (15,000×); (**M**) Mitochondria 16 h after treatment (60,000×); (**N**) Endoplasmic reticulum 24 h after treatment (40,000×); and (**O**) Cytoplasm 24 h after treatment (30,000×).

Electron microscopy showed that control columnar (col) cells in the midgut of *M. separata* larvae were arranged in order and had intact cell plasma membranes (Figure 3a). The cavosurface of the goblet (gob) cells showed regular microvilli and the mitochondria showed a complete bilayer membrane and an apparent ridge (Figure 3c). The endoplasmic reticulum was arranged neatly and had numerous ribosomes (Figure 3d). The cytoplasm was highly dense and the organelles were distributed in sequence (Figure 3e).

Six hours after treatment with PSNW, the ultrastructure of midgut cells of *M. separata* larvae showed obvious deviations compared with control cells as follows: the microvilli of the gob cells were degenerated (Figure 3g) and the apical cytoplasm was vacuolated (Figure 3i,j). After 8 h, the mitochondria were tumid and the bilayer membrane and ridge were fuzzy (Figure 3h). After 10 h, the col cells of the apical membrane microvilli expanded and degenerated, the midgut cells were disarranged, and the cell plasma membrane was compromised and severely fragmented (Figure 3f). After 12 h, the microvilli in col and gob cells degenerated partially, the cytoplasm density decreased, the cell plasma membrane was compromised and severely fragmented, and the organelles, including mitochondria, extruded outward into the lumen (Figure 3k,l). After 24 h, the mitochondria were tumid, and a part of the mitochondrial bilayer membrane disappeared and was replaced with a blank bright area (Figure 3m). The lumen of the endoplasmic reticulum showed extreme expansion and was obviously vesiculated. Spherical swelling emerged (Figure 3n) and β-type glycogenosomes were formed (Figure 3o).

#### 2.3.2. Ultrastructure Effects of PSNW on the Midgut Cells of *A. ispilon* Larvae

**Figure 4:**
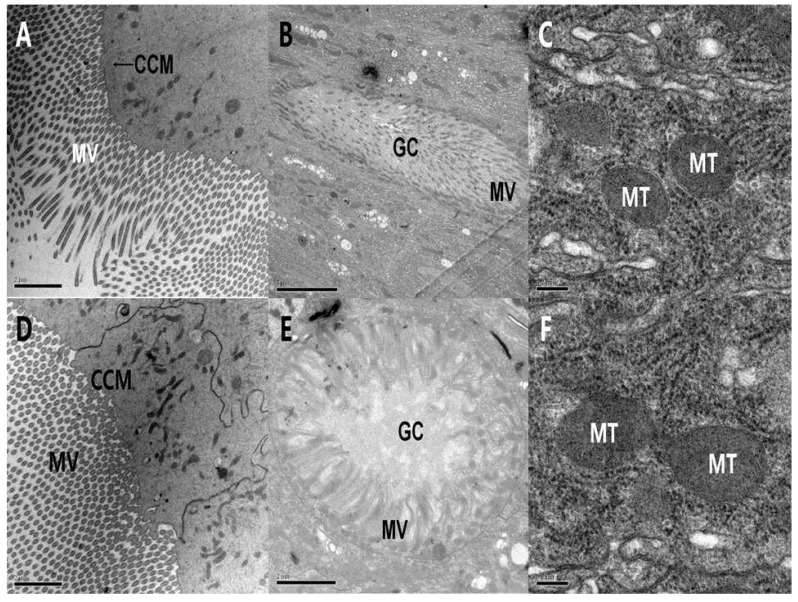
Ultrastructural effect of periplocoside NW (PSNW) on the midgut cells of *A. ispilon* larvae. (**A**) Control of columnar cell membrane and microvilli (12000×); (**B**) Control of goblet (gob) cells (8,000×); (**C**) Control of mitochondria (80,000×); (**D**) Columnar cell membrane and microvilli 24 h after treatment (12,000×); (**E**) Goblet cells after 24 h treatment (15,000×); (**F**) Mitochondria 24 h after treatment (80,000×).

Electron microscopy showed that the control col cells were arranged in order (Figure 4a), and the intercellular structure was compact (Figure 4b) in the midgut of *A. ispilon* larvae. The mitochondria bilayer membrane was clear, the cytoplasm was dense, the endoplasmic reticulum was arranged in an orderly manner, and numerous ribosomes were on the surface (Figure 4c).

Ultrastructural observations on the midgut cells of *A. ispilon* larvae after PSNW treatment showed that no obvious changes were induced by PSNW. After 24 h, col cells microvilli were arranged in order, cytoplasm was dense, and the cytoplasmic membrane was intact (Figure 4d). The gob cell microvilli were arranged in order and showed no degeneration (Figure 4e). The mitochondrial bilayer membrane and ridge in cells were clear, and the organelles and numerous ribosomes on the endoplasmic reticulum surface were also arranged in order (Figure 4f).

## 3. Discussion

Fluorescent markers are often used in scientific studies because of the high specificity of the fluorescence method. When the histochemical criteria are met, the specific fluorescence can be differentiated from other fluorescence in tissues in several ways and the chemical basis of the method is well understood [10]. The results of the fluorescence localization in this study indicated that irrespective of the fact that midgut tissues were incubated with FWN *in vitro* or *in vivo*, green fluorescence was emitted by the tissues. Further observation showed that the midgut tissues of treated insects showed degradation of the cytoplasmic membrane and overall had been seriously damaged. All of these results showed that PSNW could integrate within the midgut tissues of the *M. separata* larvae and destroy the midgut cells through the destruction of the barrier function of the gut wall. Consequently, the hemolymph entered the midgut through the damaged gut wall cells.

The midgut of insects is the main organ of the digestive tract, in which digestion and absorption occur; the wall comprises a single layer of digestive epithelium and two muscle layers (inner circular and outer longitudinal) [11]. The midgut is the main target organ for many xenobiotics, which not only include dietary substances from plants [12], but also bacterial endotoxins [13]. According to previous studies, the indirect ingestion of neem oil by prey can result in severe alterations in the midgut, such as the direct cytotoxic effects of neem oil on the midgut cells of *Ceraeochrysa claveri* (Navás) larvae [14].

To investigate the mechanism of action of PSNW further, electron microscopy experiments were conducted on the larvae of two insect species, *M. separata* and *A. ispilon.* According to our previous research, PSNW caused stomach poisoning in *M. separata* larvae, but not in *A. ispilon* larvae [8]*.* In this study, the electron microscopy results showed that PSNW could cause the degradation and destruction of microvilli in the col and gob cells in the midgut tissues of the *M. separata* larvae*.* PSNW could induce the destruction of the cell membrane structure and cause cell disintegration, which is reflected by the complete disappearance of the cytoplasmic membrane. PSNW can also lead to the expansion and vesiculation of the endoplasmic reticulum, denudation of ribosomes, swelling of mitochondria, ridge blurring, and incomplete bilayer membranes. However, no apparent changes were observed in the midgut cells of *A. ispilon* larvae treated with PSNW. We speculated that one of the intoxication mechanisms of PSNW in *M. separata* larvae was the destruction of the cell membrane, inner membrane, and organelles in the midgut*.* PSNW may therefore be a digestive poison.

The mechanism of action of *Bacillus* *thuringiensis* (Bt) δ-endotoxins and celangulin V [15,16] can serve as a reference for research on the mechanism of action of PSNW. Bt δ-endotoxins can interact with the larval midgut epithelium, causing a disruption in membrane integrity and ultimately leading to death of the insect [17,18]. The main action mechanism of the insecticidal component celangulin V might be to bind with the receptor in the midgut, change the structure of the cell membrane, and disturb the normal function of the membrane [19]. Because of the severity of structural damage of the cytomembrane, as observed in studies with Bt δ-endotoxins, the most characteristic effect of PSNW may be the change of membrane permeability, disruption of the cell’s ionic balance, and changing the cell’s osmotic pressure. Subsequently, water absorbed by the insect infiltrates the gut wall cells through the hemolymph, resulting in cell swelling and ultimately cell death. However, further experiments are needed to unravel the specific binding sites in the midgut cytomembranes of *M. separata* larvae treated with PSNW.

## 4. Materials and Methods

### 4.1. Compounds

**Figure 5:**
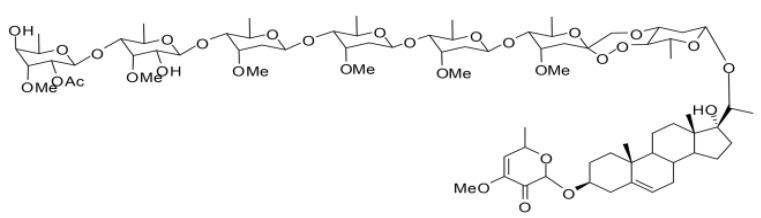
Chemical structure of periplocoside NW (PSNW).

All reagents were of analytical grade unless otherwise specified. PSNW was extracted and purified from *P. sepium* Bunge root barks at the Institute of Pesticide Science, Northwest Agriculture and Forestry University (NWAFU), Yangling, China. The chemical structure of PSNW is shown in Figure 5. Purity of PSNW is over 98% according to HPLC analysis.

### 4.2. Insects

Larvae of the 5th instar of the lepidopteran armyworm *M. separata* were provided by the Institute of Pesticide Science, NWAFU. The *M. separata* colony was maintained in the laboratory for 17 years at 25 °C, 70% relative humidity, and a photoperiod of 16 h:18 h light:dark with periodic introduction of field-collected insects.

### 4.3. Insect Treatment

Newly molted 6th instar larvae of *M. separata* and *A. ispilon* were starved for 24 h and subsequently fed with fresh wheat leaf discs (0.5 cm × 0.5 cm) coated with 1 μL of 5 mg/mL PSNW solution. The control group was fed with acetone-treated leaf discs. Twenty insects were used from each group (treated group and control group).

### 4.4. Symptoms Observation

The treated and control group insects were placed individually into Petri dishes (6 cm diameter). The poisoning symptoms were evaluated visually at the following different times: 0.5, 1, 2, 3, 4, 5, 6, 8, 10, 12, 16, 24, and 48 h. Typical symptoms were recorded with a DF-1000A single lens reflex (SLR) camera (Sea-Gull, Tianjin, China).

### 4.5. Fluorescence Localization Studies

#### 4.5.1. Fluorescence Marker of PSNW (FNW)

**Figure 6:**
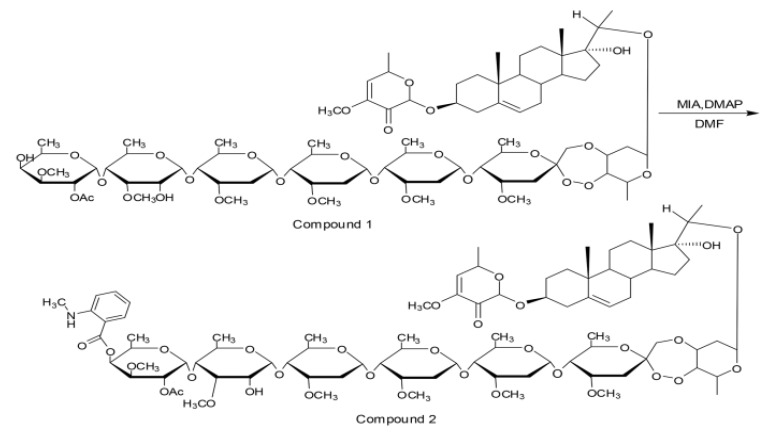
Synthesis of the fluorescent derivative MANT-PSNW.

The fluorescence marker synthesis reaction of PSNW is illustrated in Figure 6. PSNW (compound 1; 30 mg, 0.0188 mmol), isatoic acid anhydride (2.5 mg, 0.0188 mmol), and 4-demethylaminopyridine (2.3 mg, 0.0188 mmol) were placed inside dried pear-shaped bottles and weighed. These compounds were dissolved in 2 mL anhydrous N,N-dimethylformamide and, subsequently, allowed to react under magnetic stirring at room temperature. The reaction was monitored via thin layer chromatography. After completion of the reaction, a quenching reaction was performed by adding 2 mL methanol to the reaction system. After decompressing and evaporating the solvent, flash column chromatography was performed and the final product (compound 2) was obtained (Figure 6). The structure of the compound was confirmed via NMR and MS analysis and its purity was over 95% according to HPLC analysis.

#### 4.5.2. Incubation

Incubation *in vivo.* Newly molted 5th instar larvae of *M. separata* were starved for 24 h and subsequently fed with fresh wheat leaf discs (0.5 cm × 0.5 cm) soaked in 10 mg/mL FNW solution for 2 s. The control group was fed with acetone-treated leaf discs. After 24 h, all the treated insects that showed the obvious poisoning symptoms were sampled and the midgut was dissected.

Incubation *in vitro.* Newly molted 5th instar larvae of *M. separata* larvae were starved for 24 h. The midgut was dissected and incubated in 1 mg/mL FWN at 28 °C for 30 min followed by rinsing several times with phosphate buffered saline (PBS).

#### 4.5.3. Fixing and Staining

The dissected midguts were fixed with 4% paraformaldehyde for 2 to 4 h and were then dehydrated in graded sucrose solution for 24 h until all midguts settled at the bottom. Ultrathin sections (10 µm) were sliced using a freezing microtome and stained with 3 µg/mL CM-DiI (Invitrogen, California, USA) at 37 °C for 5 min and at 4 °C for 15 min. The uncombined fluorochrome was washed with PBS and the slide was sealed with 50% glycerinum phosphate buffer for confocal laser scanning microscopy (CLSM).

#### 4.5.4. CLSM

The prepared sections were observed via LSM-710 confocal laser scan microscope (CLSM) (Zeiss, Oberkochen, Germany). FNW and CM-DiI emitted green and red fluorescence under laser excitation at 405 and 550 nm, respectively. Eyepiece multiple was set to 10× and the objective multiple was adjusted to 100× and 400× for hierarchal scanning. Photomicgraphs are from a representative experiment repeated three times with similar results. 

### 4.6. Transmission Electron Microscopy (TEM)

Samples of the midgut were pre-fixed in 40 mL/L glutaraldehyde and post-fixed in 10 g/L aqueous osmium tetroxide for 30 min. Fixed samples were rinsed in 0.1 M PBS with sucrose (pH 7.4), dehydrated in graded acetone, and embedded in Epon812. Ultrathin sections were cut with a Leica-ULTRACUT (Zeiss, Oberkochen, Germany), stained with uranyl acetate and lead citrate, and examined on a JEM1230 electron microscope (Jeol, Munchen, Germany) at 80 kV. Photomicgraphs are from a representative experiment repeated three times with similar results.

## 5. Conclusions

PSNW may be a digestive poison that affects the cytomembrane in the midgut tissues of *M. separata* larvae*.* One or more binding sites for PSNW may exist on the cytomembrane of the midgut tissues of *M. separata* larvae, but this theory should be further confirmed through biotechnology methods, such as genomics, proteomics, and bioinformatics.

## Abbreviations

MV: microvilli
GC: goblet cell
CC: columnar cell
CCM: columnar cell membrane
MT: mitochondria
ER: endoplasmic reticulum
V: vacuole
Lu: midgut lumen
bl: basal lamina
al: apical lamina
gs: â-type glycogenosome
